# Potential Use of Phenolic Acids as Anti-*Candida* Agents: A Review

**DOI:** 10.3389/fmicb.2015.01420

**Published:** 2015-12-21

**Authors:** Guilherme R. Teodoro, Kassapa Ellepola, Chaminda J. Seneviratne, Cristiane Y. Koga-Ito

**Affiliations:** ^1^Oral Biopathology Graduate Program, São José dos Campos Institute of Science and Technology, Universidade Estadual PaulistaSão José dos Campos, Brazil; ^2^Oral Sciences, Faculty of Dentistry, National University of SingaporeSingapore, Singapore; ^3^Department of Environmental Engineering and Biopathology Graduate Program, São José dos Campos Institute of Science and Technology, Universidade Estadual PaulistaSão José dos Campos, Brazil

**Keywords:** *Candida*, phenolic acids, phenolic compounds, antifungal effect, synergism

## Abstract

There has been a sharp rise in the occurrence of *Candida* infections and associated mortality over the last few years, due to the growing body of immunocompromised population. Limited number of currently available antifungal agents, undesirable side effects and toxicity, as well as emergence of resistant strains pose a considerable clinical challenge for the treatment of candidiasis. Therefore, molecules that derived from natural sources exhibiting considerable antifungal properties are a promising source for the development of novel anti-candidal therapy. Phenolic compounds isolated from natural sources possess antifungal properties of interest. Particularly, phenolic acids have shown promising *in vitro* and *in vivo* activity against *Candida* species. However, studies on their mechanism of action alone or in synergism with known antifungals are still scarce. This review attempts to discuss the potential use, proposed mechanisms of action and limitations of the phenolic acids in anti-candidal therapy.

## Introduction

*Candida* species are a major group of fungal pathogens in humans, particularly among immunocompromised and hospitalized patients (Cuellar-Cruz et al., 2012). *Candida albicans* inhabits various body surfaces like oral cavity, gastrointestinal tract, vagina, and skin of the healthy individuals as a commensal organism (Kleinegger et al., 1996; Huffnagle and Noverr, 2013). Host-related factors can predispose the transformation of harmless *Candida* into an opportunistic pathogen, causing infection or candidiasis in superficial mucous surfaces which can progress into invasive mycoses (Nett and Andes, 2006). Foregoing factors include, but not limited to immuno-suppression, prolonged treatment with wide-spectrum antibiotics and chronic diseases (Kullberg and Arendrup, 2015; Polke et al., 2015). The epidemiology of invasive candidiasis varies geographically (Morgan, 2005; Pfaller et al., 2011). It significantly increases the period of hospitalization, economic burden and mortality, especially in ICU patients or those under chemotherapy or with a history of abdominal surgery (Falagas et al., 2006; Berdal et al., 2014; Drgona et al., 2014).

Only few classes of antifungals such as polyenes, azoles, echinocandins, allylamines, and flucytosine are available for the treatment of *Candida* infections (Sanglard et al., 2009). However, there are various undesirable properties, most importantly the dose-related toxicity in aforementioned antifungals (Chandrasekar, 2011). Ideally, an antifungal should have null or reduced toxicity toward human cells (Wong et al., 2014). For instance, amphotericin B is a polyene available for systemic administration, but its use has been limited due to its systemic side effects such as nephrotoxicity (Odds et al., 2003). Azole antifungals have some side effects associated with gastrointestinal, hepatic, and endocrinologic disorders and interfere with oxidative drug metabolism in the liver (Joly et al., 1992).

In addition, rising drug resistance is an inevitable problem. In particular, *Candida glabrata* and *Candida krusei* show intrinsic resistance to fluconazole, the drug of choice for AIDS patients (Kanafani and Perfect, 2008; Siikala et al., 2010; Rautemaa and Ramage, 2011). Drug resistance has already been reported for recently introduced echinocandin antifungal agents (Hakki et al., 2006; Ben-Ami et al., 2011; Clancy and Nguyen, 2011; Seneviratne et al., 2011). Moreover, biofilm mode of *Candida* is known to be highly resistant to antifungal agents (Chandra et al., 2005; Niimi et al., 2010). Therefore, it is necessary to discover new antifungal agents or safer alternatives to improve the efficacy of treatment against *Candida* infections. In this regard, antifungal agents based on natural resources, such as phenolic compounds may be an alternative strategy to negate the rising antifungal drug resistance (Negri et al., 2014). This review attempts to critically analyze the possible use of phenolic acids as a therapeutic strategy against *Candida* infections.

**FIGURE 1:**
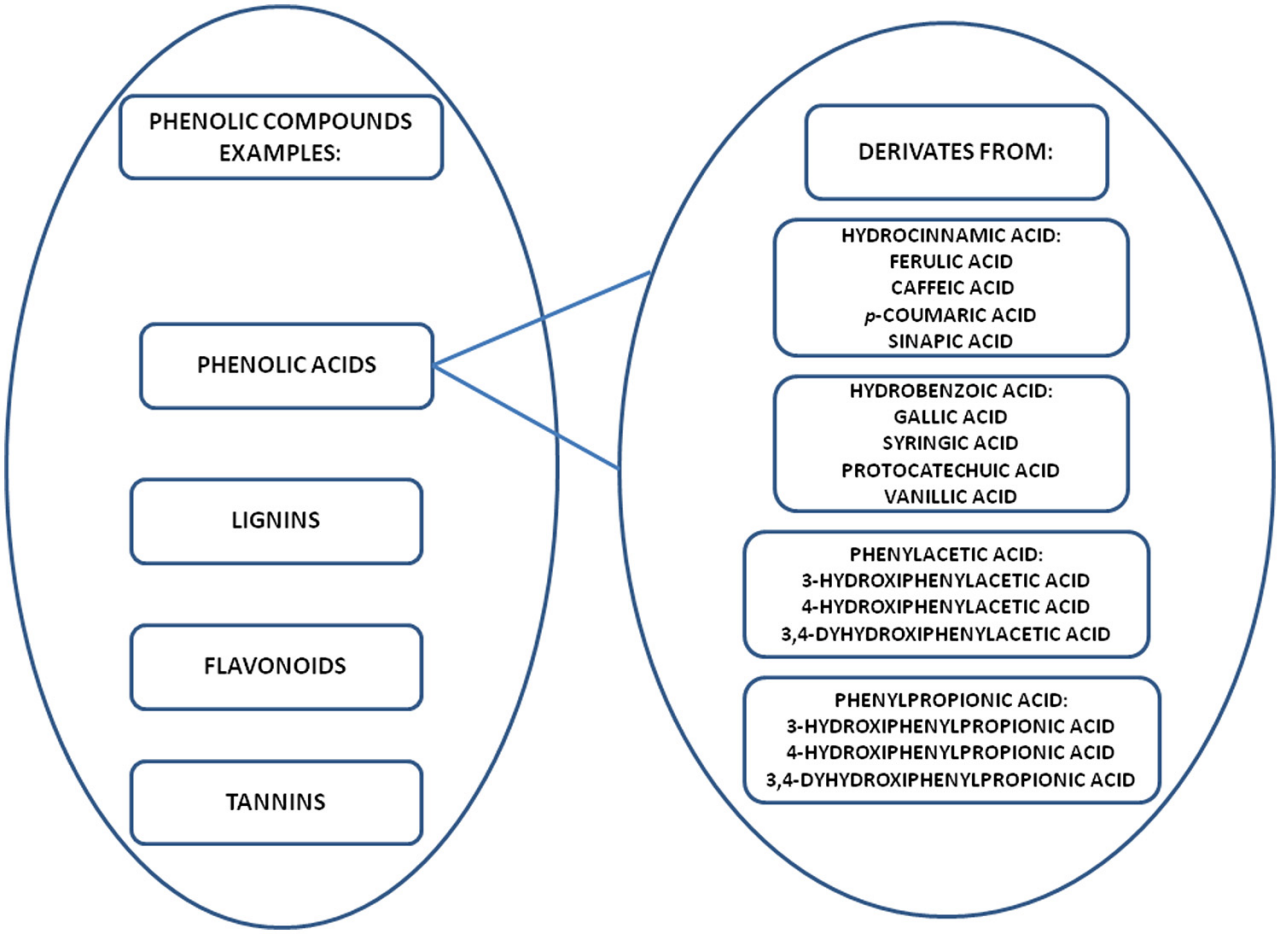
**Examples of phenolic compounds and main derivates classified as phenolic acids**.

Phenolic compounds are widely found in plant foods (fruits, cereal grains, legumes, and vegetables) and beverages (tea, coffee, fruits juices, and cocoa). The most common phenolic compounds are phenolic acids (cinnamic and benzoic acids), flavonoids, proanthocyanidins, coumarins, stilbenes, lignans, and lignins (**Figure 1**; Cowan, 1999; Chirinos et al., 2009; Khoddami et al., 2013). The anti-*Candida* properties of phenolic compounds that have been widely reported in the literature include inactivation of enzyme production (Evensen and Braun, 2009) and anti-biofilm effect (Evensen and Braun, 2009; Shahzad et al., 2014).

**FIGURE 2:**
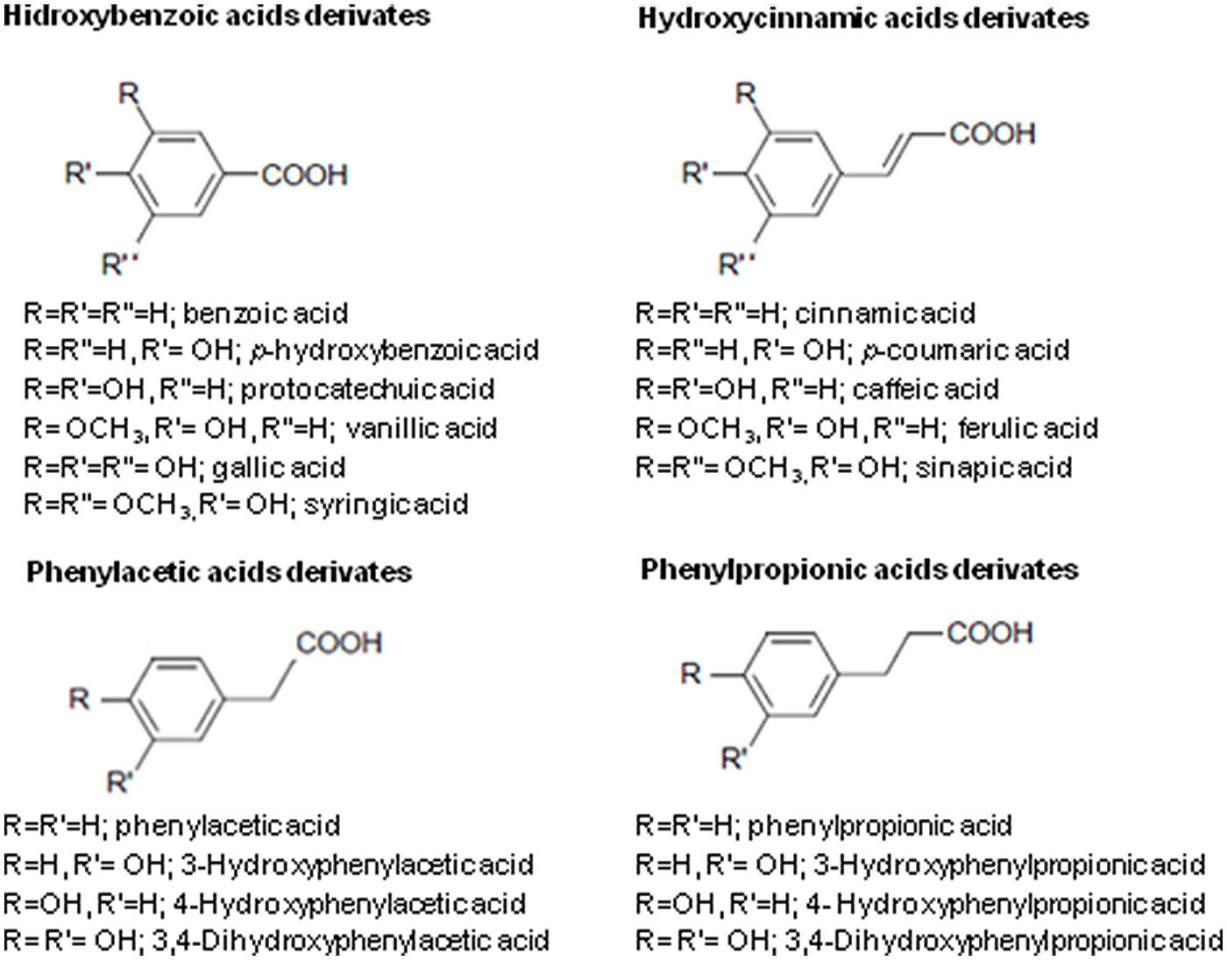
**General chemical structures of the phenolic acids [based on Pereira et al. (2009) and Cueva et al. (2010)]**.

Phenolic acids are derivatives of hydrocinnamic, hydrobenzoic, phenylacetic, and phenylpropionic acids (**Figures 1** and **2**; Pereira et al., 2009; Cueva et al., 2010). Phenolic acids commonly exist as esters, glycosides or amides in nature, but not in their free form. The determining factor for characterization of phenolic acids is the number and the location of hydroxyl groups on the aromatic ring. Some natural sources are rich in phenolic acids and shown to possess a promising action against *Candida* (**Table 1**). In this review, we discuss the anti-candidal activity of the phenolic acid compounds, possible mechanism of actions and future directions.

**Table 1:** Phenolic acids derived from plants extracts showing activity against *Candida* sp.

Plant	Phenolic acids found	Type of extract	Microorganism	MIC value μg/ml	MBC value μg/ml	Reference
*Buchenavia tomentosa*	Gallic acid	Aqueous	*C. albicans* ATCC 18804 *C. tropicalis* ATCC 13803 *C. krusei* ATCC 6258 *C. glabrata* ATCC *C. parapsilosis* ATCC 22019 *C. dubliniensis* NCPF 3108	200–12500	6500 *C. krusei* (ATCC 6258)	Teodoro et al., 2015
*Rosa rugosa*	Protocatechuic, gallic, and *p*-coumaric acids	Methanolic	*C. albicans* ATCC 10231 *C. parapsilosis* ATCC 22019	156	1250	Nowak et al., 2014
*Teucrium arduini* L.	Ferulic acid	Ethanolic	*C. albicans* ATCC 10231	4000	NR	Kremer et al., 2013
*Potentilla* sp.	Caffeic acid and ferulic acid	Acetonic and methaolic	*C. albicans* ATCC 10231	780–1560	NR	Wang et al., 2013
*Dimocarpus longan* Lour	Gallic acid	Spray-dried or Freeze-dried water	*C. krusei* ATCC 10231 *C. parapsilosis* ATCC 22019 *C. albicans* ATCC 90028 and clinical strains	500–4000	NR	Rangkadilok et al., 2012
*Ligusticum mutellina L.*	Gallic, *p*-OH-benzoic, caffeic, *p*-coumaric, and ferulic acids	Methanolic	*C. albicans* ATCC 10231 *C. parapsilosis* ATCC 22019	1250	2500	Sieniawska et al., 2013
*Limonium avei*	Caffeic, *m*-coumaric, *p*-coumaric, ferulic, isovanillic, *p*-methoxybenzoic, protocatechuic, sinapinic, and vanillic acids	Ethanolic	*C. albicans* ATCC 10231	4000	>4000	Nostro et al., 2012
*Kitaibelia vitifolia*	*p*-hydroxybenzoic, caffeic, syringic, *p*-coumaric, and ferulic acids	Ethanolic	*C. albicans* ATCC 10231	15.62	NR	Maskovic et al., 2011
*Tamarix gallica L.*	Gallic, synnapic, *p*-hydroxybenzoic, syringic, vanillic, *p*-coumaric, ferrulic, *trans*-2-hydroxycinnamic and *trans*-cinnamic acids	Hydromethanolic	*C. kefyr, C. holmii, C. albicans, C. sake, C. glabrata*	2000	NR	Ksouri et al., 2009
*Cirsium* sp.	Caffeic, *p*-coumaric, ferulic, *p*-hidroxybenzoic, protocatechuic vanillic, and gallic acids	Aqueous	*C. albicans* ATCC 10231	780–1560	6250 to >50000	Nazaruk et al., 2008
*Olea europaea L.*	Caffeic acid	Aqueous	*C. albicans* CECT 1394	5000^∗^	NR	Pereira et al., 2007
*Anogeissus latifolia*	Gallic acid	Hydroalcoholic after maceration with ether	*Candida albicans* (MTCC 183)	7.28 μg/ml	NR	Govindarajan et al., 2006
Berry (Cloudberry Raspberry, Strawberry)	Hydroxycinnamic acids	Acetonic 70%	*Candida albicans* NCPF 3179	1000 μg/ml	NR	Nohynek et al., 2006

NR, not reported; *IC_25_.

## Antifungal Activity of Phenolic Acids Against *Candida* Species

Natural extracts containing phenolic acids have demonstrated antifungal activity against *Candida* species (**Table 1**). Phenolic acid derivatives isolated from these sources such as gallic, caffeic, cinnamic, benzoic, protocatechuic, and phenylacetic acids also have antifungal activity (**Table 2**). However, the antifungal effect of the natural extracts may vary due to the differences in the quantity and the type of phenolic acid. In addition, the solvents used for extraction may also affect the antifungal effect. Moreover, other compounds present in natural extracts may act synergistically with phenolic acids to enhance the overall antifungal effect (Pereira et al., 2007; Nowak et al., 2014). Therefore, phenolic acids derived from different natural sources have highly variable MIC values against *Candida* (**Table 2**). Hence, a clear understanding of the composition of phenolic acids present in the natural extract is important to assess its potential as an antifungal agent (Salvador et al., 2004; Rangkadilok et al., 2012).

**Table 2:** Evidences from literature regarding anti-*Candida* effect of phenolic acids.

Molecule	Anti-*Candida* effect	Result found	Reference
Gallic acid	Planktonic cells of *C. albicans* (ATCC 18804), *C. krusei* (ATCC 6258), *C. parapsilosis* (ATCC 22019), *C. dubliniensis* (NCPF 3108), and *C. glabrata* (ATCC 90030)	MIC (μg/ml) respectively: 10000, 10000, 10000, 10000, 8	Teodoro et al., 2015
	Planktonic cells and biofilm of *C. albicans* (ATCC 90028), *C. glabrata* (ATCC 2001), *C. parapsilosis* (ATCC 22019), and *C. tropicalis* (ATCC 750)	MIC (μg/ml) planktonic: <156 μg/ml MIC (μg/ml) biofilm respectively: 5000, 1250, 625, 625	Alves et al., 2014
	Planktonic cells (plate diffusion)	MIC (mg cm^-3^): 2.5	Manayi et al., 2013
	Planktonic cells of *C. albicans* (ATCC 10231) and *C. tropicalis* (ATCC 750)	MIC and MFC (μg/ml) respectively: 200, 200, 200, 100	Gehrke et al., 2013
	Planktonic cells of *C. albicans* (ATCC 90028) and 5 clinical strains, *C. krusei* (ATCC 6258), and *C. parapsilosis* (ATCC 20019)	MIC (μg/ml) respectively: 4000,4000,8000,4000, 16000, 16000, 8000, 4000	Rangkadilok et al., 2012
	Planktonic cells of *C. albicans* (ATCC 10231) and *C. parapsilosis* (ATCC 22019)	MIC (μg/ml) respectively: 8, 16	Ozcelik et al., 2011
	Planktonic cells of *C. albicans* (ATCC 90028), *C. krusei* (ATCC 6258), and *C. parapsilosis* (ATCC 22019)	MIC (μg/ml): 100	Liu et al., 2009
	Biofilm of *C. albicans* (not cited strain)	MIC (μg/ml): 1000	Wang et al., 2009
	Planktonic cells of *C. albicans* (MTCC 183)	MIC (μg/ml): 1.78	Govindarajan et al., 2006
	Planktonic cells of *C. albicans* (not cited strain)	Halo: 12 mm (100 μg on a sterile filter paper disk with 6 mm diameter)	Fogliani et al., 2005
Caffeic acid	Planktonic cells of *C. albicans* and inhibition of isocitrate lyase activity assay	MIC (μg/ml): 1000; inhibition of 91,5% of the isocitrate lyase enzyme activity	Cheah et al., 2014
	Planktonic cells and biofilm of *C. albicans* (ATCC 10231)	MIC (μg/ml): planktonic: 128; pre-formed, 4 and 24 h biofilm: 256	De Vita et al., 2014
	Planktonic cells of *C. albicans* (ATCC 10231) and *C. parapsilosis* (ATCC 22019)	MIC (μg/ml) respectively: 8, 16	Ozcelik et al., 2011
Protocatechuic acid	Planktonic cells of *C. albicans* (LMP709U)	MIC and MFC (μg/ml) respectively: 156, 312	Kuete et al., 2009
	Planktonic cells of *C. albicans* (10231) and *C. tropicalis* (ATCC 7349)	MIC (μg/ml) respectively: 500, 400	Pretto et al., 2004
Phenylacetic acid	Planktonic cells (plate diffusion) of *C. albicans* (clinical strains)	Halo: 8–10.5 mm (20 μl of a 2000 ng/ml phenylacetic acid water solution on sterile filter paper disk with 6 mm diameter)	Mendonca Ade et al., 2009
Cinnamic acid	Immunoregulatory effect on monocytes activation against *C. albicans* (SC 5314)	Significant reduce of *C. albicans* counts in 50 and 100 μg/ml	Conti et al., 2013
	Planktonic cells of *C. albicans* (ATCC 90028, ATCC 10231, PYCC 3436T) *C. parapsilosis* (ATCC 22019, PYCC 2545), *C. glabrata* (PYCC 2418T) *C. tropicalis* (PYCC 3097T), *C. krusei* (PYCC 3341), *C. lusitaniae* PYCC 2705T and synergism with antifungals	IC 50 (mmol l^-1^): 0.09 to 0.74; none synergism found	Faria et al., 2011
Benzoic acid	Planktonic cells of *C. albicans* (ATCC 90028, ATCC 10231, PYCC 3436T) *C. parapsilosis* (ATCC 22019, PYCC 2545), *C. glabrata* (PYCC 2418T) *C. tropicalis* (PYCC 3097T), *C. krusei* (PYCC 3341), *C. lusitaniae* PYCC 2705T and synergism with antifungals	IC 50 (mmol l^-1^): 0.05–0.73 Synergism found to *C. albicans* with amphotericin and itraconazole	Faria et al., 2011

The main *Candida* virulence factors are exoenzymes production, biofilm formation, adherence, and dimorphism (Vuong et al., 2004; Netea et al., 2008; Williams et al., 2011). Few studies have demonstrated the influence of phenolic acids against these factors. Anti-biofilm effect of phenolic acids against *Candida* sp. was reported (Wang et al., 2009; Alves et al., 2014; De Vita et al., 2014). However, the studies used only reference samples or did not cite the tested strain (**Table 2**). The anti-biofilm effect of these molecules should be carried out with clinical isolates *in vitro* and *in vivo*, since the ultimate goal of using these molecules is to treat candidiasis and a wider range of strains could provide more reliable results. Besides that, it also has found an influence of caffeic acid derivate against the *Candida* dimorphism (Sung and Lee, 2010).

However, several studies described effect on *Candida* virulence factors of some others phenolic molecules. For instance, bisbibenzyl stimulates the synthesis of farnesol, an inhibitor of hyphae formation, via upregulation of *Dpp3* gene (Zhang et al., 2011). Hence, bisbibenzyl may reduce *C. albicans* hyphal formation and affect biofilm formation. Moreover, anti-hyphae effect in *C. albicans* was also found following the treatment with epigallocatechin-gallate (Han, 2007), licochalcone A, gladribin (Messier and Grenier, 2011), and thymol (Braga et al., 2007). Additionally, eugenol reduces germ tube formation in *C. albicans* (Pinto et al., 2009). Beyond that, several studies have shown anti-biofilm (Messier et al., 2011; Alves et al., 2014; Rane et al., 2014; Shahzad et al., 2014) and anti-adhesive (Feldman et al., 2012; Rane et al., 2014; Shahzad et al., 2014) activities of phenolics against C*andida*.

The number of studies on other phenolic molecules on *Candida* virulence factors with interesting results inspires a carefully investigation of phenolic acids influence on these factors.

## Mechanism of Action, Biological Pathways, and Synergism with Antifungal Agents of Phenolic Acids Against *Candida*

**FIGURE 3:**
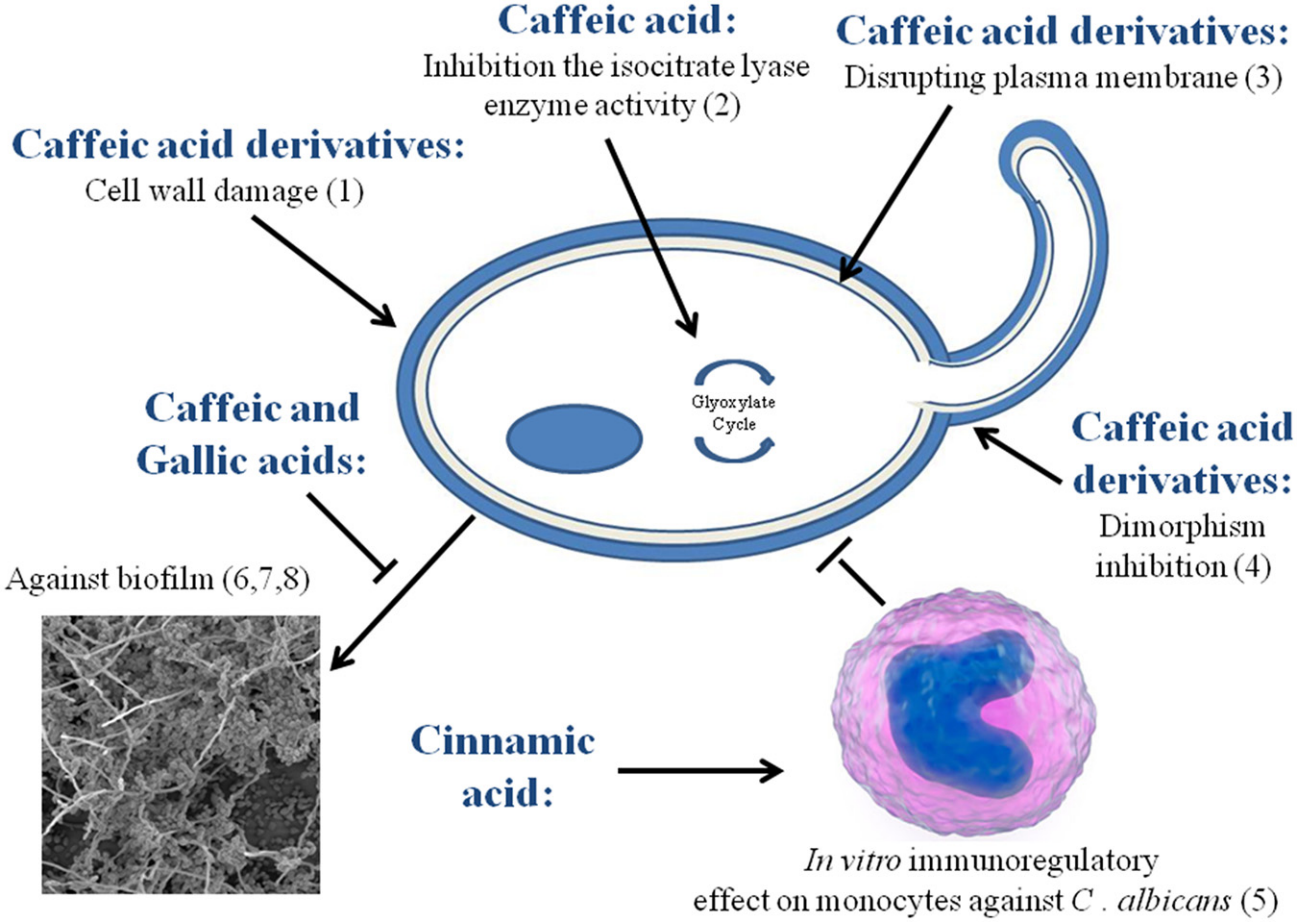
**Described mechanisms of action and biological pathways of some phenolic acids against *Candida*.** (1) Ma et al. (2010); (2) Cheah et al. (2014); (3,4) Sung and Lee (2010); (5) Conti et al. (2013); (6) Alves et al. (2014); (7) De Vita et al. (2014); (8) Wang et al. (2009).

In order to obtain some insights on the antifungal activity of phenolic acids, herein we compare the existing data along the lines of mechanism of action, synergy with known antifungal agents and others biological pathways (**Figure 3**).

### Mechanisms of Action and Biological Pathways

Phenolic acids such as ferulic and gallic acids are known to affect the cell membrane of Gram-positive and Gram-negative bacteria leading to a change in cell surface hydrophobicity and charge, ultimately causing leakage of cytoplasmic content (Borges et al., 2013). A similar effect has been suggested for the caffeic acid derivative on *Candida* cytoplasmatic membrane (Sung and Lee, 2010). Furthermore, a possible effect on the *C. albicans* cell wall has been shown for caffeic acid derivatives which may interfere with 1,3-β-glucan synthase (Ma et al., 2010).

It is noteworthy that polyene antifungals also cause pouring of cellular contents through direct binding to ergosterol, distorting the membrane function. Also, azole antifungal agents inhibit biosynthesis of ergosterol (Vanden Bossche et al., 2004). No study on the effect of phenolic acid on the ergosterol composition or biosynthesis could be detected.

Mode of action of several others phenolic compounds provide some clues to deduce the mechanism of phenolic acids. For instance, isoquercetin (Yun et al., 2015), curcumin (Lee and Lee, 2014), and lariciresinol (Pinto et al., 2009) can damage the *C. albicans* cell membrane. On the other hand, eugenol and methyleugenol cause considerable reduction in the ergosterol biosynthesis in *Candida* and subsequently affecting the cell membrane (Ahmad et al., 2010b). Similar effect has been observed with epigallocatechin-3-gallate (Navarro-Martinez et al., 2006), thymol and carvacrol (Ahmad et al., 2011). Besides, cardanol demonstrated chitin-binding ability in *C. albicans* cell wall (Mahata et al., 2014).

Few studies have found about others biological pathways of phenolic acids against *Candida*. Exemplifying, an *in vitro* immunoregulatory effect on monocytes against *C. albicans* by cinnamic acid (Conti et al., 2013) and a inhibition of *C. albicans* isocitrate lyase enzyme activity after treatment with caffeic acid (Cheah et al., 2014) was reported. However, several studies have suggested that the other biological pathways and cellular targets of others phenolic compounds may be different from that of existing antifungal agents. Some phenolic compounds have shown to induce apoptotic mechanisms in *Candida*, thereby contributing to their antifungal activity (Zore et al., 2011). For instance, eugenol inhibits the cell cycle at G1, S, and G2-M phases in *C. albicans* and consequently induces apoptosis. Another phenolic compound, curcumin also induces apoptosis in *C. albicans*, by increasing the reactive oxygen species (ROS) and induction of *CaMCA1* gene expression (Cao et al., 2009). On the contrary, baicalein increases ROS causing perturbation in mitochondrial homeostasis in *C. krusei* without inducing apoptosis (Kang et al., 2010). Methyl chavicol seemed to induce aptotosis in *C. albicans* although the exact pathway is still not clear (Khan et al., 2014). Blocking effect of thymol, carvacrol (Ahmad et al., 2013) and baicalein (Huang et al., 2008) on the drug transporter pumps in *Candida* has been demonstrated using rhodamine 6G dye. Inhibition of eﬄux transporters results in accumulation of antifungal compounds inside the cell making *Candida* highly susceptible to the antifungal agent (Huang et al., 2008). These helpful anti-*Candida* biological pathways observed for phenolic molecules, mainly on the drug transporters pumps may contribute to elucidate the possible effects of phenolic acids against *Candida*.

Another aspect to be considered is that previous studies reported that some *Candida* species were able to metabolize phenolic acids (Middelhoven et al., 1992; Middelhoven, 1993). *C. parapsilosis* was able to grow in the presence of some phenolic acids after 3 days of cultivation. On the other hand, *C. tropicalis* was unable to grow in the presence of phenolic acids even after 14 days of cultivation (Middelhoven, 1993). These evidences should be better investigated in the future. Further studies are warranted to obtain a deeper understanding of the mechanism of action and others biological pathways of phenolic acids on *Candida* cells.

### Synergism with Existing Antifungal Agents

Apart from rising antifungal resistance, there are other important limitations in the existing antifungal agents, such as inadequate spectrum of activity, poor bioavailability, small tolerance index, interactions with other drugs, inadequate pharmacokinetic profile, and considerable toxic effects (Lewis and Graybill, 2008; Pfaller et al., 2010). Although phytochemicals remain an important source for the discovery of new antifungal agents, micro-plate based *in vitro* screening assays have not shown higher effectiveness of plant extracts when compared to the existing antifungal agents with higher efficacy (Newman and Cragg, 2012). Hence, in general, plant extracts with higher minimum inhibitory concentrations (MICs) such as 1000 μg/ml are considered ineffective (Morales et al., 2008).

Therefore, some studies have explored the possibility of synergistic activity of phenolic acids and existing antifungal agents in order to maximize the antifungal effect. It is a good strategy to study the synergistic effect when MIC values of phenolic acids against *Candida* are highly variable (Rauha et al., 2000; Kalinowska et al., 2014). Synergistic effect of benzoic acid with amphotericin B and itraconazole against *C. albicans* has been reported in literature (Faria et al., 2011; **Table 3**). However, mechanism of this synergistic effect of phenolic acids and conventional antifungal agents is poorly understood. Therefore, it is important to examine similar synergistic effects shown by others phenolic compounds and conventional antifungal agents in order to obtain some insight.

**Table 3:** Synergism of phenolic compounds with traditional antifungals in their action against *Candida albicans.*

Compound	Fluconazole	Amphotericin B	Itraconazole	Others
2,5 Dihydroxybenzaldehyde	___	Faria et al., 2011	Faria et al., 2011	___
Baicalein	Huang et al., 2008^†^	Fu et al., 2011	___	___
Benzoic acid^∗∗^	___	Faria et al., 2011	Faria et al., 2011	___
Benzyl benzoate	Zore et al., 2011^†^	___	___	___
Butylated hydroxyanisole	Simonetti et al., 2002^†^	Andrews et al., 1977^∗^; Beggs et al., 1978^∗^		Simonetti et al., 2003^†^
Carvacrol	Ahmad et al., 2013^‡^	___	___	___
Cinnamaldehyde	Khan and Ahmad, 2012	___	___	___
Curcumin I	Sharma et al., 2010^‡^	Sharma et al., 2010	Sharma et al., 2010^‡^	Sharma et al., 2010^‡^
Epigallocatechin-gallate	Hirasawa and Takada, 2004^‡^	Hirasawa and Takada, 2004^‡^; Han, 2007	Navarro-Martinez et al., 2006	Navarro-Martinez et al., 2006
Eugenol	Ahmad et al., 2010a^‡^; Zore et al., 2011; Khan and Ahmad, 2012^∗∗∗^	___	___	___
Glabridin	Liu et al., 2014	___	___	Messier and Grenier, 2011
Honokiol	Jin et al., 2010^†^	___	___	___
Licochalcone A	___	___	___	Messier and Grenier, 2011
Methyleugenol	Ahmad et al., 2010a^‡^	___	___	___
Punicalagin	Endo et al., 2010^†^	___	___	___
Propyl gallate	D’Auria et al., 2001^†^	Andrews et al., 1977; Beggs et al., 1978^∗^	D’Auria et al., 2001^†^	Strippoli et al., 2000^†^
Thymol	Guo et al., 2009^‡^; Faria et al., 2011; Ahmad et al., 2013^‡^	Guo et al., 2009; Faria et al., 2011	Faria et al., 2011	___

*^∗^Ineffectiveness antifungal effect of phenolic alone; ^∗∗^phenolic acid; ^∗∗∗^performed on biofilm formation; ^†^resistant strain; ^‡^resistant and susceptible strains.*

A promising synergism between phenolic compounds and fluconazole against resistant strains of *Candida tropicalis* was described recently (da Silva et al., 2014). Several other studies have also demonstrated a significant synergism between other known antifungals and phenolic compounds against *C. albicans* (**Table 3**). Some studies suggested that the synergism is due to the induction of apoptosis by an increase in the production of ROS. Hence, it was found that amphotericin B together with baicalein or curcumin increases the production of ROS (Sharma et al., 2010; Fu et al., 2011). A similar effect has been observed with fluconazole and curcumin (Sharma et al., 2010).

Another hypothesis for the aforementioned synergism is the association between folic acid cycle and ergosterol biosynthesis pathways of *C. albicans*. Hence, epigallocatechin-gallate, a phenolic compound was demonstrated to have a synergistic antifungal effect on *Candida* when combined with itraconazole or ketoconazole (Navarro-Martinez et al., 2006). Azoles directly inhibit the ergosterol biosynthesis while epigallocatechin-gallate has an antifolatic effect that indirectly affects the ergosterol biosynthesis. Epigallocatechin-gallate causes a depletion of the enzyme S-adenosylmethionine which in turn affects the enzyme Sterol C24 methyltransferase. Hence, lower production of C24 methyltransferase negatively affects the ergosterol biosynthesis. Direct and indirect effects on ergosterol biosynthesis explain the synergism between epigallocatechin-gallate and azoles (Navarro-Martinez et al., 2006).

Another study has shown that phenolic compounds such as thymol and carvacrol significantly decrease the expression levels of virulence genes *CDR1* and *MDR1* in fluconazole-resistant *C. albicans* (Ahmad et al., 2013). An *in vivo* study on systemic candidiasis in mice demonstrated that following the treatment with honokiol and fluconazole, the survival rate was 100% while a monotherapy showed only a survival rate of 80% to fluconazole and 20% to honokiol, respectively. Furthermore, the synergism of these two compounds led to a notable reduction in *C. albicans* counts in mouse kidneys compared with the fluconazole treatment alone (Jin et al., 2010). Similarly, mice treated with epigallocatechin-gallate and amphotericin B survived approximately 24 and 30 days longer when compared to the groups treated only with epigallocatechin-gallate or amphotericin B, respectively (Han, 2007). Considering the foregoing evidence obtained for other phenolic compounds, it is likely that potential of synergism exists between known antifungal agents and phenolic acids and this possibility needs to be examined in future.

### Safety of the Phenolic Acids *In vitro* and *In vivo*

An ‘ideal’ antifungal agent for *Candida* infections should not have side effects or toxicity (Chapman et al., 2008; Wong et al., 2014). However, in reality, all the antifungals currently in use have some side effects on gastrointestinal tract, liver and kidney (Wingard et al., 1999; Bates et al., 2001). Therefore, practically one would expect to have some dose-related side effects from any new antifungal agent. It is imperative to understand this limitation in order to appreciate promising qualities of the drug under investigation. DNA-damaging effect of phenolic acids has been observed in p53R cell lines treated with gallic acid (Hossain et al., 2014). Moreover, *in vivo* hepatotoxicity was observed in rats when given a diet supplemented with more than 200 mg/kg/day of gallic acid (Galati et al., 2006). In addition, hematological disorders, as well as liver and kidney weight increase were observed in rats fed with 0.6–5% of gallic acid daily for 13 weeks (Niho et al., 2001).

A potential carcinogenicity was observed on the fore-stomach of rats when fed with a powdered diet containing 0.4% of caffeic acid for up to 28 weeks (Hirose et al., 1998). The clastogenic power of caffeic and cinnamic acids have been described *in vitro* (Maistro et al., 2011). Subcronic administration of protocatechuic acid (0.1% in drinking water) for 60 days has shown a possible liver and kidney toxicity in mice (Nakamura et al., 2001). Sodium benzoate and sodium phenylacetate have been used in the treatment of acute hyperammonaemia and are derived from benzoic acid and phenylacetic acid respectively. Inappropriate doses of both substances may cause plasma acidosis, hypotension, cerebral edema and other neurotoxical effects, sometimes even death of patients (Kaufman, 1989; Praphanphoj et al., 2000). Phenylacetic acid can also affect the osteoblastic functions *in vitro* and increase cell proliferation in the alveolar region (Kaufmann et al., 2005; Yano et al., 2007). Sodium and potassium benzoates could be clastogenic, mutagenic and cytotoxic to human lymphocytes *in vitro* (Zengin et al., 2011). Therefore, is imperative to examine the dose-related toxicity of phenolic acids in a series of comprehensive *in vitro*, *in vivo* and clinical studies before administration as an antifungal agent.

## Conclusion

Phenolic acids demonstrate considerable antifungal properties against *Candida*. Previous studies have shown phenolic acid compounds possess considerable anti-adhesion, anti-biofilm effects, and inhibitory activity on morphogenesis and exoenzyme production of *Candida* species. However, hitherto no clear mechanism of action of phenolic acids on *Candida* cells and virulence factors has been described compared to the existing antifungal agents. Interestingly, there is substantial evidence of the synergistic effect of phenolic acids and existing antifungal agents which may become a promising anti-candidal strategy. However, more studies are in demand for a conclusive statement regarding their role. Therefore, we propose that more comprehensive studies are mandatory to obtain evidence regarding the suitability of the use of phenolic acids as a successful antifungal agent in future.

## Author Contributions

GT conceived, designed, did the literature review, provided and wrote the manuscript. KE assisted in the preparation, design, final review, and co-wrote the manuscript. CK-I and CS conceived, designed, assisted in the literature and final review, and co-wrote the manuscript.

## Conflict of Interest Statement

The authors declare that the research was conducted in the absence of any commercial or financial relationships that could be construed as a potential conflict of interest.
